# Whole-genome informed circulating tumor DNA analysis by multiplex digital PCR for disease monitoring in B-cell lymphomas: a proof-of-concept study

**DOI:** 10.3389/fonc.2023.1176698

**Published:** 2023-06-02

**Authors:** Zahra Haider, Tove Wästerlid, Linn Deleskog Spångberg, Leily Rabbani, Cecilia Jylhä, Birna Thorvaldsdottir, Aron Skaftason, Hero Nikdin Awier, Aleksandra Krstic, Anna Gellerbring, Anna Lyander, Moa Hägglund, Ashwini Jeggari, Georgios Rassidakis, Kristina Sonnevi, Birgitta Sander, Richard Rosenquist, Emma Tham, Karin E. Smedby

**Affiliations:** ^1^ Department of Molecular Medicine and Surgery, Karolinska Institutet, Stockholm, Sweden; ^2^ Department of Medicine, Division of Clinical Epidemiology, Karolinska Institutet and Karolinska University Hospital, Stockholm, Sweden; ^3^ Department of Hematology, Karolinska University Hospital, Stockholm, Sweden; ^4^ Department of Clinical Genetics, Karolinska University Hospital, Stockholm, Sweden; ^5^ Clinical Genomics Stockholm, Science for Life Laboratory, School of Engineering Sciences in Chemistry, Biotechnology and Health, Royal Institute of Technology, Stockholm, Sweden; ^6^ Department of Oncology-Pathology, Karolinska Institutet, Stockholm, Sweden; ^7^ Department of Clinical Pathology and Cancer Diagnostics, Karolinska University Laboratory, Stockholm, Sweden; ^8^ Department of Laboratory Medicine, Division of Pathology and Cancer Diagnostics, Karolinska Institutet and Karolinska University Hospital, Stockholm, Sweden; ^9^ Genomic Medicine Center Karolinska, Karolinska University Hospital, Stockholm, Sweden

**Keywords:** cell-free DNA (cfDNA), whole genome sequence (WGS), liquid biopsy, lymphoma, measurable (minimal) residual disease (MRD), droplet digital (ddPCR), CtDNA, IGH-BCL2 translocation

## Abstract

**Introduction:**

Analyzing liquid biopsies for tumor-specific aberrations can facilitate detection of measurable residual disease (MRD) during treatment and at follow-up. In this study, we assessed the clinical potential of using whole-genome sequencing (WGS) of lymphomas at diagnosis to identify patient-specific structural (SVs) and single nucleotide variants (SNVs) to enable longitudinal, multi-targeted droplet digital PCR analysis (ddPCR) of cell-free DNA (cfDNA).

**Methods:**

In 9 patients with B-cell lymphoma (diffuse large B-cell lymphoma and follicular lymphoma), comprehensive genomic profiling at diagnosis was performed by 30X WGS of paired tumor and normal specimens. Patient-specific multiplex ddPCR (m-ddPCR) assays were designed for simultaneous detection of multiple SNVs, indels and/or SVs, with a detection sensitivity of 0.0025% for SV assays and 0.02% for SNVs/indel assays. M-ddPCR was applied to analyze cfDNA isolated from serially collected plasma at clinically critical timepoints during primary and/or relapse treatment and at follow-up.

**Results:**

A total of 164 SNVs/indels were identified by WGS including 30 variants known to be functionally relevant in lymphoma pathogenesis. The most frequently mutated genes included *KMT2D*, *PIM1*, *SOCS1* and *BCL2*. WGS analysis further identified recurrent SVs including t(14;18)(q32;q21) (*IGH::BCL2*), and t(6;14)(p25;q32) (*IGH::IRF4*). Plasma analysis at diagnosis showed positive circulating tumor DNA (ctDNA) levels in 88% of patients and the ctDNA burden correlated with baseline clinical parameters (LDH and sedimentation rate, p-value <0.01). While clearance of ctDNA levels after primary treatment cycle 1 was observed in 3/6 patients, all patients analyzed at final evaluation of primary treatment showed negative ctDNA, hence correlating with PET-CT imaging. One patient with positive ctDNA at interim also displayed detectable ctDNA (average variant allele frequency (VAF) 6.9%) in the follow-up plasma sample collected 2 years after final evaluation of primary treatment and 25 weeks before clinical manifestation of relapse.

**Conclusion:**

In summary, we demonstrate that multi-targeted cfDNA analysis, using a combination of SNVs/indels and SVs candidates identified by WGS analysis, provides a sensitive tool for MRD monitoring and can detect lymphoma relapse earlier than clinical manifestation.

## 1 Introduction

Mature B-cell lymphomas are a heterogenous group of lymphoid neoplasms affecting blood and lymphatic tissues. More than 80 different subtypes of lymphomas have been described by the World Health Organization (WHO) and International Consensus Classification (ICC), based on immunophenotype, histopathology and genomic aberrations of the malignant cells (1, 2). In addition to the biological and molecular diversity, lymphomas also display heterogeneity in the clinical course of the disease, ranging from indolent to aggressive forms. The most common B-cell lymphoma subtypes include diffuse large B-cell lymphoma (DLBCL) and follicular lymphoma (FL), accounting for 30-40% and 15-20% of newly diagnosed lymphoma cases, respectively (3, 4). Despite improvements in the clinical management of DLBCL and FL (5, 6), a considerable number of patients do not respond to therapy and/or relapse after initial treatment response (7, 8). Lack of primary treatment response and/or early relapse within two years of primary diagnosis is associated with poor overall survival (8, 9).

Current risk stratification of lymphoma patients at diagnosis and choice of treatment are guided by clinical and pathological parameters, such as age, serum lactate dehydrogenase (LDH) levels, performance status (ECOG) score, Ann Arbor stage and extranodal sites (ENS). Immunohistochemistry and fluorescence *in situ* hybridization (FISH) analyses are also performed, when clinically indicated, to characterize recurrent chromosomal events such as *BCL2* or *MYC* translocations that have diagnostic and prognostic impact in DLBCL and FL (1, 2).

Novel molecular classification schemes with prognostic potential have been proposed for risk stratification of patients at diagnosis. In FL, the prognostic impact of gene mutations has been shown in the m7-FLIPI model, which stratified FL patients into low-risk and high-risk following first-line immunochemotherapy (10). In DLBCL, the cell-of-origin (COO) classification characterizes patients into transcriptome-based subgroups, one resembling germinal center B-cells (GCB) and the other resembling activated B-cells (ABC or non-GCB subgroup), while genetic classification stratifies patients in up to 7 subtypes with prognostic significance based on the diverging somatic mutational landscapes (10–15). While these molecular classifiers were included in the recently updated WHO as well as ICC classifications (1, 2, 9–15), none of them are currently applied in routine practice to guide treatment decisions. In addition, a proportion of DLBCL cases remain unclassified, further highlighting the complex molecular heterogeneity of the disease (9). Therefore, additional methods including comprehensive molecular diagnostics are needed to improve the risk stratification in lymphomas which would lead to more effective therapeutic strategies and might enable efficient implementation of targeted therapies.

Patient outcome can be further improved by utilizing sensitive methods to measure treatment response and measurable residual disease (MRD) during primary treatment as well as for early detection of refractory disease and/or relapse. Traditionally, imaging scans by computed tomography (CT) and positron emission tomography (PET) are used for evaluating MRD and response to treatment in DLBCL and FL. Though their non-invasiveness and sensitivity are beneficial, these methods have the limitations including their incapacity to measure dynamic clonal changes. Analyzing circulating cell-free DNA (cfDNA) in plasma has emerged as a promising tool for monitoring MRD in many cancers including lymphomas (16–19). In healthy individuals, cfDNA fragments are released into circulation by apoptotic cells. However, in cancer patients, a proportion of cfDNA is composed of DNA shed from the tumors, designated as circulating tumor DNA (ctDNA). The mutational profile of ctDNA reflects the aberrant genomic profile of the tumor tissue of origin and can be characterized by the tumor-specific aberrations, including single nucleotide variants (SNVs), small insertions and deletions (indels), large structural variants (SVs) and copy-number variants (CNVs) (20, 21). Thereby, longitudinal cfDNA analysis of ctDNA burden enables tracking of residual disease at a molecular level and, owing to its minimal invasiveness, facilitates multiple serial sampling during the course of the disease (22).

In this study, we aimed to assess the clinical potential of whole-genome sequencing (WGS) in molecular diagnostics and utilized genomic aberrations characterized at diagnosis to design sensitive, patient-specific multiplex ddPCR (m-ddPCR) assays for analyzing plasma cfDNA and monitoring treatment response and MRD. We performed this proof-of-concept in a pilot cohort within the prospective BioLymph study (ISRCTN12948913) aimed at improving diagnosis and outcome in patients with lymphoma.

## 2 Materials and methods

### 2.1 Patient cohort

The present study included 9 patients from the prospective study BioLymph (ISRCTN12948913) that consecutively recruits all newly diagnosed patients with lymphoma at the Department of Hematology, Karolinska University Hospital, Sweden, since February 2019.

**Figure 1:**
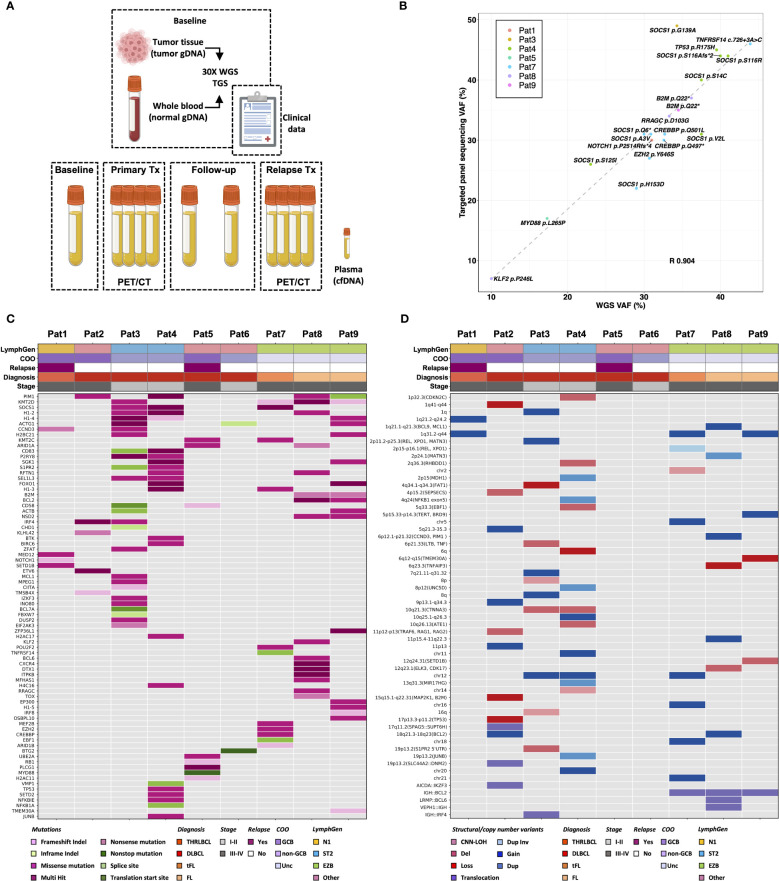
Comprehensive genomic profiling of diagnostic lymphoma tissue. **(A)** Summary of sample collection and baseline analyses performed in the study. Genomic profiling of 9 lymphoma patients at diagnosis was performed by targeted gene-panel sequencing and 30X whole-genome sequencing (WGS) using genomic DNA from matching tumor and normal tissue. **(B)** WGS analysis was compared with targeted gene panel sequencing analysis performed within BioLymph study. Variant allele frequency (VAF) of 20 clinically relevant SNVs/indels, identified in 7/9 patients by targeted gene panel sequencing, correlated positively with VAF detected by 30X WGS (R, Pearson correlation coefficient). **(C)** Mutational spectrum of 164 somatic SNVs/indels identified in 78 genes (rows) by WGS in lymphomas patients (columns) at diagnosis. **(D)** Landscape of large SVs characterized by WGS at baseline, including deletions (del), tandem duplications (dup), inversions (inv) and inter- and intrachromosomal translocations. Additionally, CNVs such as gains, losses, and copy-number neutral loss of heterozygosity (CNN-LOH), detected by WGS analysis are shown, annotated with chromosomal bands and potentially implicated genes in parenthesis. To correlate somatic genomic aberrations with biological and clinical lymphoma subtypes, patients are annotated with Ann Arbor staging, cell-of-origin (COO) classification, relapse status, and classification based on LymphGen genetic classifier (14). THRLBCL, T-cell/histocyte rich large B-cell lymphoma; DLBCL, diffuse large B-cell lymphoma; tFL, transformed follicular lymphoma; FL, follicular lymphoma; GCB, germinal center B-cell like; Unc, unclassified; gDNA, genomic DNA; cfDNA, cell-free DNA; TGS, targeted gene-panel sequencing.

After obtaining written consent from patients, fresh-frozen tumor tissue biopsies and matching peripheral blood samples for germline analysis were collected at diagnosis according to the study protocol, along with the baseline clinical information (**Figure 1A**). Radiology was performed according to clinical routine: CT imaging at interim evaluation and PET-CT at end of treatment (final evaluation) and subsequently when clinically indicated. Patient selection for the present study was based on material availability, morphological diagnosis and for three patients, presence of recurrent translocations detected by FISH analysis of diagnostic tumor tissue in clinical routine.

The study was approved by the Stockholm Regional Ethical committee (2017/2538-31) and conducted in accordance with the Declaration of Helsinki.

### 2.2 Cell-free plasma collection

The BioLymph study entailed plasma sample collection at diagnosis, after first cycle of the primary treatment, at interim evaluation, at final evaluation and during follow-up (once per year for 2 years) (**Figure 1A**). In the event of relapse, the study protocol included collection of plasma samples before start of relapse treatment, at interim and after end of relapse treatment (**Figure 1A**).

Peripheral blood samples for cell-free plasma isolation were collected in Cell-Free DNA BCT tubes (STRECK, USA) and stored at room temperature for a maximum of 7 days. Cell-free plasma was extracted from blood by centrifugation (1 600 ×g for 10 minutes at 4°C), followed by a second centrifugation (16 000 ×g for 10 minutes at 4°C). The plasma was aliquoted and stored at −80°C.

### 2.3 Cell-free DNA isolation

Cell-free plasma from patients (2-8 ml for diagnostic samples and 3-4ml for follow-up samples) and plasma from healthy blood donors (for normal cfDNA) was thawed and cfDNA was extracted using the QIAamp Circulating Nucleic Acid Kit™ (Qiagen) on a QIAvac24 Plus vacuum manifold, according to the manufacturer’s protocol. CfDNA was eluted in 40 ml AVE buffer and stored at −20°C until further analysis.

### 2.4 Normal genomic DNA extraction

Peripheral blood from an anonymous group of 10 non-cancer patients was collected in EDTA tubes (BD Vacutainer^®^) and pooled. QIAamp DNA Blood Maxi kit (Qiagen) was used for gDNA extraction, according to the manufacturer’s protocol. DNA was eluted in 1 ml buffer AE, after which an additional 1 ml buffer was added and eluted to maximize gDNA yield. The eluates were pooled and frozen at -20°C.

### 2.5 Library preparation for whole-genome sequencing

Libraries using 1100 ng gDNA for WGS were prepared with TruSeq DNA PCR-Free (Illumina, San Diego, CA, US) for all patients except for Pat5 and Pat9. Due to limited sample availability, the libraries for Pat5 and Pat9 were prepared with 200 ng input gDNA using NxSeq AmpFREE Low DNA Library Kit (Lucigen, Biosearch Technologies, UK). Briefly, gDNA was fragmented with Covaris E220 (Covaris, MA, US) to obtain insert size of approximately 350 base pairs (bp). The fragments were end-repaired and a-tailed, followed by ligation of unique dual index adaptors (Illumina). Library quantification was performed by KAPA Library Quantification Kit (Roche) and sequencing was done on a NovaSeq 6000 system (Illumina) using paired-end 150 bp readout, aiming at 300 million (M) paired reads.

### 2.6 Bioinformatic analyses of whole-genome sequencing

Somatic SNV/indel, SV and CNV calling from raw FASTQ files generated by WGS was performed by Bioinformatic Analysis pipeLine for SomAtic MutatIons in Cancer (BALSAMIC) version (v) 10.0.2 (23) and IgCaller (v1.3) (24). Demultiplexing of reads from the sequencer was performed using bcl2fastq2 Conversion Software v2.20 (Illumina). Detailed descriptions of somatic variant calling, filtering and classification are provided below:

#### 2.6.1 Somatic variant calling

BALSAMIC version (v) 10.0.2 (23) was used to analyze the data from raw FASTQ files generated by WGS. First, quality of FASTQ files was controlled using FastQC v0.11.9. Adapter sequences and low-quality bases were trimmed using fastp v0.23.2 (25). Trimmed reads were mapped to the reference genome hg19 using sentieon-tools (26). The resulting SAM files were converted to BAM files and sorted using samtools v1.15.1 (27). Duplicated reads were marked using Picard tools MarkDuplicate v2.27.1 and promptly quality controlled using CollectMultipleMetrics and CollectWgsMetrics functionalities. Results of the quality-controlled steps were summarized by MultiQC v1.12 (28). Somatic SNVs/indels were called for each sample using Sentieon TNscope and TNhaplotyper (29). The called variants were further filtered using the following criteria: total depth (tumor, normal) ≥10, alternate allele depth (tumor) ≥3, allele frequency (AF) (tumor) ≥0.05, Maximum AF (tumor)<1, GNOMADAF_popmax ≤0.001, normalized base quality scores ≥20 and read counts of alternate and reference alleles >0. SVs were called using Manta v1.6.0 (30), Delly v1.0.3 (31) and TIDDIT v3.0.0 (32). CNVs were called using ascatNgs v4.5.0 (33) and Delly v1.0.3 (31). The SV calls from Manta, Delly, TIDDIT and ascatNgs (tumor-normal) were merged using SVDB v2.6.0 (32). All variants were finally annotated using Ensembl VEP v104.3 (34). We used vcfanno v0.3.3 (35) to annotate somatic SNVs for their population AF from gnomAD v2.1.1 (36). For immunoglobulin (IG) gene rearrangements, paired tumor and normal BAM files from WGS were further analyzed by IgCaller v1.3 (24).

#### 2.6.2 Somatic variant filtering and classification

Somatic SNVs/indels were filtered to retain potential protein-altering lymphoma-associated variants (**Figure S1**). Briefly, variants annotated as exonic, splicing and present in 252 genes included in the GMS Lymphoid panel were retained. The functional relevance of variants was assessed using the Molecular Tumor Board Portal (MTBP) v7.0 (37). Briefly, variants were functionally classified as either putatively functional, putatively neutral or of unknown significance (VUS). The putatively functional variants were then matched for biomarkers reported in lymphoid neoplasms and clinical actionability was described as tiers according to a modified ESMO-ESCAT scale (38).

Impact prediction of splice acceptor and donor variants was performed by Alamut™ Visual Plus (SOPHiA GENETICS™, Saint Sulpice, Switzerland) and SpliceAI (39). Variants with no predicted impact or predicted impact <40% by Alamut™ and/or delta score ≤0.5 by SpliceAI were classified as either neutral or VUS, depending on the classification assigned by MTBP. Variants with significant predicted impact and/or delta score >0.5 were classified as potentially functional regardless of the MTBP classification. Splice region variants such as *BCL2* c.-294C>G with no apparent truncating affect in a MANE transcript by manual inspection was classified as VUS rather than functionally relevant despite having significant predicted impact and delta score.

SVs (i.e., tandem duplications, inversions, deletions, and translocations) were filtered from SVDB file to retain SVs larger than 1000 bp, implicating protein-coding regions and detected by more than one SV caller relying on discordant read pairs and/or split reads (Manta, DellySV and Tiddit) (30–32) (**Figure S1**). SVs with total paired reads ≤20 and with discordant paired reads and/or split reads making ≤15% of total paired reads spanning the breakpoints were filtered out. SVs were manually analyzed by visualizing the breakpoints in BAM files using IGV v2.11.1.

CNV analysis was performed by interpreting tumor and germline raw ASCAT profiles, segmented LogR and segmented B-allele frequency (BAF) plots (**Figure S2**). Chromosomes with germline LogR at 0 and germline BAF with three bands at 1, 0 and ~0.5 were considered valid. CNVs were defined with clearly distinct increased or decreased LogR and corresponding changes in BAF or ASCAT raw profiles.

### 2.7 LymphGen classification

Patients were classified using the NCI LymphGen 2.0 classifier (14) accessed here: https://llmpp.nih.gov/lymphgen/index.php.

### 2.8 Targeted gene panel sequencing

WGS analysis results were compared to results from targeted gene panel sequencing of lymphoma samples performed within BioLymph study protocol. This entailed panel sequencing of gDNA from fresh-frozen lymphoma and matched germline samples using an amplicon-based, customized TruSight lymphoma panel including 43 genes (Illumina) on a MiSeq (Illumina) between 2019-2021. From 2021, a broader capture-based gene panel, the Genomic Medicine Sweden Lymphoid Gene Panel (GMS Lymphoid), including 252 genes (Twist Bioscience, CA, US) (40) was employed. Library preparation, sequencing and bioinformatic analyses for targeted GMS Lymphoid panel were performed as recently described (40, 41). Libraries were sequenced on a NovaSeq 6000 (Illumina) instrument using paired-end 150 bp readout, at a depth of 20 M read pairs per sample. From both panels, SNVs/indels with variant allele frequency (VAF) of ≥5% in 43 genes included in the TruSight panel were manually ranked according to the ACMG criteria (42).

### 2.9 Droplet digital PCR

#### 2.9.1 Droplet digital PCR assay design

ddPCR assays for SNVs/indels, with probes labelled with 5’-FAM™ or 5’-HEX™ fluorophores and 3’-Iowa Black^®^ Fluorescent Quencher (FQ), were obtained from Bio-Rad (Bio-Rad, CA, US) (**Table S6**). Custom ddPCR assays for SVs, copy-number reference (CNR) assay Albumin and 1 SNV (*IRF4* p.L24V) were designed using Primer3Plus web interface (Whitehead Institute for Biomedical Research, Massachusetts Institute of Technology), according to Bio-Rad guidelines (**Table S7**). Dark probes i.e., probes labelled with only 3’-Iowa Black^®^ FQ, were designed to target the wild-type allele for some Bio-Rad ddPCR assays using the MIQE context sequence of the corresponding Bio-Rad assay (**Table S6**). Custom designed primers and HPLC purified probes, with 5’-FAM™ or 5’-HEX™ fluorophores and 3’-Iowa Black^®^ Fluorescent Quencher, and HPLC purified, were ordered from IDT (Integrated DNA Technologies) (**Table S7**). Locked nucleic acid (LNA) bearing Affinity Plus^®^ probe was ordered for *IRF4* p.L24V (**Table S7**).

#### 2.9.2 Droplet digital PCR

ddPCR analysis was performed, as previously described (43), using the QX200 AutoDG Droplet Digital PCR System (Bio-Rad, California, USA) according to manufacturer’s protocol. For a singleplex ddPCR, a reaction mix of 22 μl was prepared with 1X of 4X ddPCR Multiplex Supermix for Probes (no dUTP) (Bio-Rad), 1X FAM and HEX ddPCR assays and template DNA. For amplitude multiplex ddPCR (m-ddPCR), optimal assay concentrations of unique assays intended for multiplexing were added (range 0.3X-1.5X) (**Table S6**). After droplet generation using QX200 AutoDG (Bio-Rad), PCR amplification was performed in SimpliAmp™ Thermal Cycler (ThermoFisher, MA, US) with the following program: 95°C for 10 minutes, 40 cycles of 94°C for 30 seconds and 55°C or 60°C for 60 seconds, and 98°C for 10 minutes followed by an infinite hold at 4°C. The ramp rate was set at 2°C/second for each step, except 1°C/second for holding step. Data acquisition was performed in QX200 Droplet reader (Bio-Rad).

#### 2.9.3 Singleplex and multiplex ddPCR optimization

Optimal annealing temperature (Tm), assay specificity and amplitude were assessed for all assays following Bio-Rad Rare Mutation Detection Best Practices Guidelines, as previously described (43, 44). Characteristics of each assay were assessed using gradient ddPCR (Tm 55-65°C) in singleplex and multiplex reactions using 10 ng gDNA from diagnostic tumor tissue (positive control), 10 ng normal gDNA (negative control), and nuclease-free water as non-template control (NTC).

For optimizing amplitude m-ddPCR, adjustment in assay concentrations for each individual assay in a multiplex setting was guided by the amplitude characteristics of the assays in a singleplex setting. Assay concentration combinations were tested to ensure representation of each target as a distinct cluster when visualized in a 2D amplitude plot. Annealing temperatures for m-ddPCR assays were further optimized by gradient ddPCR to improve positive cluster discrimination.

Patient cfDNA samples were analyzed using optimized m-ddPCR protocols (**Table S6**) in triplicates along with positive control gDNA and NTC. Additionally, all plasma samples were analyzed with 9-12 wells with normal cfDNA for false positive rate (FPR) detection.

#### 2.9.4 Droplet digital PCR data analysis

The ddPCR data analysis was performed in QuantaSoft^TM^ Analysis Pro software v1.0.596 (Bio-Rad) following the Bio-Rad Rare Mutation Detection Best Practices Guidelines, as previously described (43). Thresholds to define positive and negative clusters were manually set by visualizing 1D and 2D amplitude plots of control wells. VAF of each target in m-ddPCR reactions was quantified by dividing the merged concentration (copies/20µl) of target by the sum of merged concentration of target plus the merged concentration of the corresponding wild-type assay. For SV targets and targets analyzed without a corresponding wild-type assay, VAF was measured by dividing the merged concentration (copies/20µl) of target with the sum of merged concentration of the other target analyzed in the same m-ddPCR with a corresponding wild-type assay plus the merged concentration of the wild-type assay. Alternatively, VAF was quantitated by CNR assays if analyzed in the same m-ddPCR reaction (**Table S6**). The FPR for each assay was designated as the VAF of target detected in normal cfDNA wells. Patient cfDNA samples were called positive if following criteria were fulfilled in merged wells: the VAF of the target analyzed was above the FPR for the target assay; the 95% CI Poisson error bars of concentration were non-overlapping between normal plasma and patient cfDNA sample; and at least three positive droplets were observed in total with at least 1 single positive droplet.

DNA copies per ml plasma were calculated, as previously described (43). First, the elution volume was divided by the product of total sample volume used for ddPCR analysis and volume of plasma. Then, the result was multiplied with the concentration of target, wild-type or CNR measured in the wells.

### 2.10 Statistical analyses

Linear correlation was measured using Pearson correlation test in R v4.1.1. and p-values ≤0.01 were considered statistically significant.

## 3 Results

### 3.1 Patient cohort

The present study included 9 patients from the prospective study BioLymph. Five of the included patients were diagnosed with DLBCL, one as a T-cell/histiocyte-rich large B cell lymphoma (THRLBCL), two patients with FL and one patient with transformed FL (tFL) (**Tables 1**, **S1**). Recurrent *IGH::BCL2* rearrangements were confirmed in the 3 latter cases by FISH analysis (**Table S1**).

**Table 1:** Clinical characteristics of patient cohort.

Clinical variable	Categories	No. of patients
Diagnosis	DLBCL	5
	THRLBCL	1
	FL	2
	tFL	1
Sex	Male	7
	Female	2
Age	≥60 years	7 (avg. 67, range 62 - 75)
	<60 years	2 (avg. 43, range 40 - 47)
ECOG	0	6
	≥1	3
Ann Arbor staging	I-II	3
	III-IV	6
ENS	Yes	1
	No	8
Treatment	R-CHOP/R-CHOEP14 x6	7
	R-Bendax6	2
Relapse	Yes	2
	No	7
B-symptoms	Yes	2
	No	7
Bulky disease (>7 cm)	Yes	3
	No	6
LDH (µkat/L)	Low <4	5
	High ≥4	4
COO	Non-GCB	3
	GCB	3
	Unclassified	3

BM, Bone marrow; COO, Cell-of-Origin; DLBCL, Diffuse large B-cell lymphoma; ECOG, Performance status; ENS, Extranodal sites; FL, Follicular lymphoma; GCB, Germinal center B-cell like; LDH, Lactate dehydrogenase; R-Benda, R Bendamustin; R-CHOP, rituximab, cyclophosphamide, doxorubicin, vincristine, prednisone; R-CHOEP, R-CHOP + etoposide; THRLBCL, T -cell/histiocyte rich large B cell lymphoma; tFL, Transformed FL.

Patients with DLBCL, THRLBCL and tFL (n=7) were treated with R-CHOP/R-CHOEP14 x6, while the 2 patients with FL received R-Bendamustin x6 (**Tables 1**, **S1**). Interim evaluation by CT after primary treatment cycle 3 showed partial remission (PR) in 5 patients. All 9 patients had complete metabolic response (CMR) as measured by PET-CT after end of primary treatment, except for one patient (Pat8) whose primary treatment had to be interrupted due to toxicity (**Table S1**).

Two patients, Pat1 and Pat5 (both classified as non-GCB DLBCL), had relapsed disease at 28 and 32 months after end of primary treatment, respectively (**Tables 1**, **S1**). Pat9 (FL) had a marginal growth after end of primary treatment, with PET-CT imaging measuring a tumor mass of 14mm in cervical glands. However, Pat9 remained asymptomatic and has not required any further treatment as of last follow-up (**Table S1**). Also, as of last follow-up, only 1 patient (Pat8) was deceased of other cause (**Table S1**).

### 3.2 Genomic profiling of lymphomas at diagnosis

For comprehensive genomic profiling, WGS analysis was performed on paired tumor/normal samples from the 9 patients with a median depth of 37X (range: 27-50X) (**Table S2**). WGS analysis identified in total 2037 SNVs/indels in exonic or splicing regions (range: 23–502) (**Figure S1**). To focus on mutations potentially relevant for lymphomas, protein altering SNVs/indels were selected in the coding regions of the 252 genes included in the GMS Lymphoid panel, known to be recurrently mutated in different lymphoid malignances (40, 45) (**Figure S1**). After filtering and variant classification, 164 SNVs/indels in 78 genes were retained (**Table S3**). These 164 variants included 20 SNVs/indels, previously detected by targeted gene panel sequencing within the BioLymph study, with matching VAFs (Pearson correlation coefficient R 0.904, p-value<0.01) (**Figures 1B, C**; **Table S3**).

While the most recurrently mutated genes were *KMT2D* and *PIM1* (44%) (**Figure 1C**), mutations in *B2M, BCL2* and *NSD2* occurred only in patients with FL (n=2) (**Figure 1C**). The variant classification and interpretation strategy via the Molecular Tumor Board Portal (37) ranked 30 SNVs/indels as functionally relevant and with likely clinical potential in lymphoma diagnostics and risk stratification (**Figure S1**; **Table S3**). These variants included recurrent alterations known in DLBCL and FL pathogenesis such as *MYD88* p.Leu265Pro (n=1), *CCND3* p.Gln276* (n=1), ETV6 c.33 + 1G>A (n=1), *CIITA* c.274dup (n=1), *CREBBP* p.Gln497* (n=1), *B2M* p.Gln22* (n=2), *KMT2D* c.14075+2T>G (n=1), *EZH2* p.Tyr646Ser (n=1) and *SOCS1* c.343del (n=1) (**Table S3**).

A comprehensive analysis of large SVs, including inter- and intrachromosomal translocations, deletions, tandem duplications, and CNV, was also performed (**Figures 1D**, **S1**; **Tables S4**, **S5**). WGS analysis validated the t(14;18)(32;21) (*IGH::BCL2*) translocations in the 3 FL/tFL patients (**Tables S4**, **S5**). Each patient had a unique combination of breakpoints in the *IGH* and *BCL2* genes (**Tables S4**, **S5**); Pat8 had a *BCL2* breakpoint in the major breakpoint cluster region (MBR) in 3’UTR, while Pat7 and Pat9 had *BCL2* breakpoints in the intermediate cluster region (ICR), almost ~14 kilobase pairs (kb) downstream of *BCL2* 3’UTR (46).

Additionally, WGS analysis identified known recurrent translocations, such as t(12;17)(p13.31;q12) (*AICDA::IKZF3*) (n=1), t(6;14)(p25.3;q32.33) (*IRF4::IGH*) (n=1) and t(3;12)(q27.3;p12.1) (*LRMP::BCL6*) (n=1) (**Figure 1D**; **Tables S4**, **S5**). Frequently occurring CNVs included gains in 1q (n=5), 2p (*REL, XPO1*) (n=4) and 18q21 (*BCL2*) (n=2) as well as losses in 6q (n=3) (**Figures 1D**, **S2**). Furthermore, CNV analysis showed aneuploidy in Pat2 with ploidy estimation of 4.07 and heterozygous loss of 17p encompassing the *TP53* gene (**Figure S2**).

Genetic classification of patients was performed using the LymphGen classifier tool (14, 15) based on SNVs/indels, CNVs and SVs (**Figures 1C, D**). The LymphGen classification subgrouped the 3 FL/tFL as “EZB”, and 2 GCB DLBCL patients as “ST2”, while Pat1 (non-GCB DLBCL) carried a *NOTCH1* mutation and was subgrouped as “N1”. The remaining three patients (Pat2, Pat5 and Pat6) were classified as “other” (**Figures 1C, D**).

### 3.3 Design and optimization of multiplex droplet digital PCR assays

For analyzing cfDNA isolated from plasma at diagnosis and follow-up, patient-specific and sensitive m-ddPCR assays were optimized for 8 patients. Targets for longitudinal MRD analysis were selected based on high VAF (according to WGS) to reflect high cancer cell fraction. Patients with variants having at least 10% VAF and with at least one of the variants known as a functionally relevant aberration in lymphoma, were chosen for plasma analysis (**Figure S3**; **Table S6**). Pat6, a patient with stage I DLBCL, had variants of unknown significance with VAFs <10% and therefore was not included in the plasma analysis (**Table S3**).

**Figure 2:**
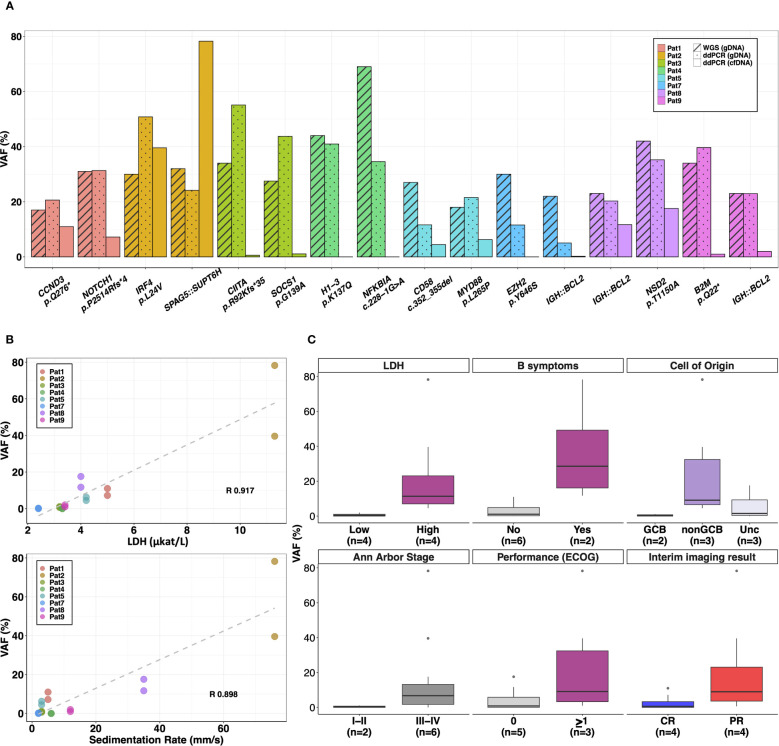
Plasma cfDNA analysis using personalized multiplex ddPCR assays. A total of 16 targets were selected to quantify ctDNA levels or VAF (%) in plasma cfDNA samples from 8 patients. **(A)** The barplots compare the VAFs (%) of the targets detected in diagnostic plasma cfDNA (analyzed by m-ddPCR) with VAF (%) in diagnostic tumor tissue (quantified by WGS and m-ddPCR). **(B)** Positive significant correlation (p-value<0.01) was observed between VAFs (%) in diagnostic plasma levels and baseline serum lactate dehydrogenase (LDH) levels as well as erythrocyte sedimentation rate. **(C)** Correlation of VAFs (%) in diagnostic plasma cfDNA with baseline clinical features and interim CT imaging results. R, Pearson correlation coefficient; GCB, germinal center like; Unc, Unclassified; CR, complete remission; PR, partial remission; n, number of patients.

For each patient, 2 targets, including SNVs, indels and SVs, were selected to design ddPCR assays (**Tables S6**, **S7**). SV ddPCR assays, including assays targeting *IGH::BCL2* translocations in 3 FL/tFL patients, were designed using patient-specific breakpoint sequences elucidated by WGS analysis (**Tables S4**, **S5**, **S7**). A total of 16 ddPCR assays (9 SNVs, 3 indels and 4 SVs) were optimized and employed for m-ddPCR analysis to quantify ctDNA levels in plasma cfDNA samples (**Figures 2A**, **S3**; **Table S6**). m-ddPCR enabled simultaneous detection of up to 3 targets per channel, including CNR assays and corresponding wild-type assays for VAF estimation (**Figure S3**; **Table S6**). Average detection sensitivity was on 0.0025% FPR for SV ddPCR assays and 0.02% FPR for SNVs/indel ddPCR assays (**Table S6**).

### 3.4 Diagnostic plasma ctDNA levels in relation to baseline clinical features and interim evaluation by CT

The average concentration of cfDNA in pre-treatment plasma from 8 patients was 36.7 ng/ml (range 0.22–123.7 ng/ml) and the total cfDNA copies/ml plasma analyzed by m-ddPCR were on average 6360 (range, 494–18852 copies/ml) (**Figure S4**). CtDNA was detected in 7/8 patients at diagnosis with an average VAF of 12.9% (range 0.07–78.2%) (**Figure 2A**). CtDNA was not detected in Pat4 with stage I DLBCL despite targets selected for plasma analysis having an average VAF of 37.5% in tumor tissue (**Figure 2A**).

Of the clinical parameters measured at diagnosis, VAF in plasma showed significant positive correlation with serum LDH levels (R 0.91, p-value<0.01) and erythrocyte sedimentation rate (R 0.90, p-value<0.01) (**Figure 2B**). When correlating categorical clinical parameters, higher concentration of total cfDNA copies/ml and VAF were observed in lymphomas of Ann Arbor stages III-IV with an average of 8306 cfDNA copies/ml and an average VAF of 17.2% compared to an average of 521 cfDNA copies/ml and 1.9% VAF in stage I-II lymphomas (**Figures 2C**, **S5**). Patients classified as GCB also had lower VAF in plasma (average 0.4%) and total cfDNA copies (average 521 copies/ml) compared with non-GCB (average 9542 cfDNA copies/ml and average VAF of 24.4%) and unclassified patients (average 7070 cfDNA copies/ml and average VAF of 6.4%) (**Figures 2C**, **S5**). Similar trends were observed with elevated average cfDNA copies/ml and VAF in plasma from patients with B-symptoms and performance status (ECOG) ≥1 (**Figures 2C**, **S5**).

After three cycles of primary treatment, interim imaging by CT showed PR in 4 patients. Both cfDNA copies/ml and VAF in plasma at diagnosis were higher in these patients (average ~7438 copies/ml and ~19.9% VAF) compared to patients with CR (average ~5281 copies/ml and ~3.56% VAF) (**Figures 2C**, **S5**).

### 3.5 Longitudinal plasma analysis detects early relapse and is more sensitive than radiological imaging

**Figure 3:**
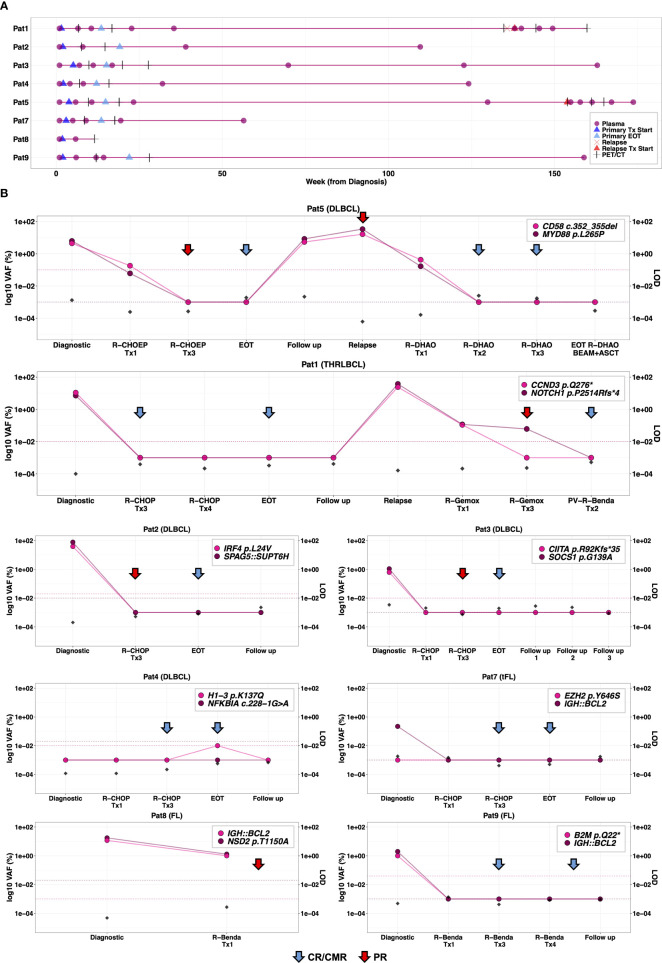
Longitudinal plasma cfDNA analysis for monitoring treatment response and surveillance. **(A)** Serial plasma collection in 8 patients analyzed by m-ddPCR analysis during clinically relevant timepoints including before primary treatment, during treatment, end of primary treatment and follow-up (once per year for two years). In the event of relapse, plasma samples were taken at relapse, during and after relapsing treatment. **(B)** Correlation of radiological evaluations with dynamics of ctDNA burden in serially collected plasma samples analyzed by m-ddPCR assays, targeting two aberrations per patient. False positive rate (FPR), measured by analyzing 9-12 wells of normal cfDNA with corresponding ddPCR assay, is plotted as horizontal dashed lines. Theoretical limit of detection (LOD) of m-ddPCR analysis (dividing 3 by total cfDNA copies analyzed) at each timepoint is plotted on the right y-axis. For plotting, 0%VAF and FPR were converted to 0.001%. During primary treatment, radiological evaluations were conducted by CT imaging at interim evaluation and PET-CT at final evaluation for all patients. CT imaging was performed at relapse for Pat1 and Pat5, as well as at interim evaluation in Pat1. All other occasions were evaluated by PET-CT. Tx, treatment cycle; EOT, end of treatment; PR, partial remission; CR, complete remission; CMR, complete metabolic remission; PET, positron emission tomography; CT, computed tomography scan.

The potential of plasma analysis for longitudinal monitoring of treatment response, and follow-up as well as for detecting relapse was investigated using serially collected plasma samples. In addition to the 8 pre-treatment samples described above, an additional 38 plasma samples were collected during treatment and follow-up from the 8 patients and analyzed by patient-specific m-ddPCR assays (**Table S6**; **Figure 3A**).

Longitudinal plasma collection was according to study protocol in Pat3, Pat4 and Pat7 (**Figure 3A**). Pat1 and Pat2 did not have plasma samples post primary treatment cycle 1 (**Figure 3A**). Also, the second follow-up sample from Pat1 was not collected in year 2. Plasma sampling for Pat5 during relapse treatment was more frequent while Pat8 plasma sampling was interrupted due to skin toxicity and respiratory complications (**Figure 3A**; **Table S1**). In Pat9, plasma sample at final evaluation of primary treatment was not available (**Figure 3A**).

There was a rapid clearance of ctDNA levels below detection threshold in 3 of 5 patients (Pat3, Pat7, Pat9) with plasma samples available after primary treatment cycle 1 and with positive ctDNA levels at diagnosis (**Figure 3B**). Pat8 (stage III FL) and Pat5 (stage III DLBCL) both had detectable ctDNA levels after cycle 1 with VAFs 1-1.2% and 0.06-0.18%, respectively (**Figure 3B**). The 6 patients with positive ctDNA levels at diagnosis and plasma samples available at interim evaluation (after cycle 3), showed complete ctDNA clearance by interim evaluation (**Figure 3B**). Of these 6, CT showed PR in 3 (Pat5, Pat2 and Pat3) (**Figure 3B**). Of the 6 patients with plasma samples available at end of primary treatment, none had detectable ctDNA levels at final evaluation of primary treatment, which correlated with PET-CT results (**Figure 3B**).

Amongst the follow-up samples, ctDNA levels were detected in Pat5 in the plasma sample taken 2 years after end of primary treatment which was 25 weeks before clinical manifestation of relapse (**Figure 3B**). Furthermore, plasma analysis could detect ctDNA burden (*MYD88* p.L265P at 0.014% VAF and *CD58* c.352_355del at 0.17% VAF) after the first cycle of second-line relapse treatment. Follow-up plasma samples taken up to ~1.5 months after completed relapse treatment (R-DHAOx3+BEAM+ASCT) remained negative correlating with PET-CT evaluations (**Figure 3B**).

In the other patient with relapse, Pat1, plasma samples were collected at relapse and after first and third R-Gemox cycle, after which treatment was switched to Polatuzumab Vedotin Benda (PV-R-Benda). The last plasma sample was collected after 2 cycles of PV-R-Benda as the patient was in CR. Increased ctDNA levels were detected at relapse and became negative after 2 cycles of PV-R-Benda, concordant with the PET-CT results showing CMR (**Figure 3B**).

## 4 Discussion

To implement precision medicine and targeted therapy approaches to improve lymphoma patient outcomes, it is essential to refine diagnostic strategies for patient classification and risk stratification. To this end, targeted NGS gene panels have been developed (40, 47). However, the biological complexity and heterogeneity of lymphoid neoplasms can make it difficult to design unified targeted panels that include genomic regions significant for all subtypes of lymphoid neoplasms. Also, designing hybridization probes for large and complex structural variants can be challenging especially when SV breakpoints are in low complexity genomic regions with repetitive sequences. In routine clinical diagnostics, detecting recurrent SVs is primarily performed using low resolution and low throughput methods such as FISH analysis. These methods are largely employed to target known SVs, thus hampering the discovery of novel rearrangements and/or translocation partners. Microarrays have been widely used to detect somatic CNVs; however, they cannot detect balanced rearrangements such as translocations, inversions, and insertions.

WGS allows characterization of all genomic aberrations, including SNVs, indels, CNVs and SVs, and has been successfully implemented in clinical diagnostics of rare inherited diseases and more lately in diagnostics of acute leukemias and pediatric cancers (48–50). To assess the clinical utility of WGS in lymphoma diagnostics, we performed 30X WGS to comprehensively profile all types of genetic aberrations in a series of 9 lymphoma patients at diagnosis. Genomic profiling at diagnosis showed that the mutational landscape in our cohort was consistent with previously reported genomic profiles of B-cell lymphomas (12, 13, 15, 19, 51). Importantly, WGS could reproduce all VAFs of the pathogenic variants that were previously detected using targeted gene panel sequencing, hence validating its reliability to detect SNVs (26, 42). WGS further identified 142 SNVs/indels in lymphoma genes, 30 of which had a known functional and potential clinical relevance according to the clinical decision support system the Molecular Tumor Board Portal (MTBP) (37).

The WGS approach employed in this study also enabled detection of breakpoints in non-coding regions resulting in identification of clinically relevant translocations, such as the *IGH::BCL2* translocation identified in 3 FL/tFL patients. WGS analysis additionally identified chromosomal translocations known to be recurrent in lymphoma pathogenesis and relevant for lymphoma subtyping, namely *IGH::IRF4* and *BCL6* rearrangements (1, 9). Therefore, by using WGS, breakpoints occurring beyond coding regions could be identified, that would be otherwise challenging to detect using gene panels or exome sequencing.

Though large wealth of data generated by WGS can also pose a challenge for downstream analysis such as variant filtering and classification, focusing variant analysis in specific genes implicated in lymphoma pathogenesis, as performed in this study, considerably reduces the need for extensive bioinformatic filtering pipelines. For CNV and SV analysis, multiple analytical tools are established for genome-wide profiling and we integrated a number of these tools in our study (30–33).

Multiple whole-genome and exome studies have defined prognostically relevant genetic subtypes of DLBCL based on mutational profiles, grouping cases with shared biological mechanisms, and providing new tools to resolve heterogeneity in lymphoma diagnostics (12–15, 52). These classifications rely on a combination of SNVs/indels, SVs and CNVs, further emphasizing the need for whole-genome characterization of diagnostic samples. Using the LymphGen classification tool in our cohort, 6/9 patients (~66%) were classified into distinct genetic subgroups (14, 15). Amongst the three unclassified patients, we could not detect characteristic genomic or structural alterations in tumor tissue in Pat6, a stage I DLBCL, despite the lymph node biopsy showing extensive, diffuse infiltration of large neoplastic lymphoid cells. Pat2, an aneuploid DLBCL with *TP53* deletion and *ETV6* mutation, had genomic features overlapping both “A53” and “MCD” subtypes and was therefore classified in the “other” group by the LymphGen tool (14, 15). Pat5, with the *MYD88* p.Leu265Pro variant, characteristic of the “MCD” group, was also unclassified; this is in line with a previous study reporting that 83% of patients with only *MYD88* mutation and no concurrent *CD79B* mutation were classified in the “other” subtype (53). The robustness and the clinical value of the genetic classification based on WGS analysis will have to be further refined and validated in subsequent studies with larger patient cohorts.

In our study, we also demonstrate a clinically feasible approach using m-ddPCR for simultaneous detection of both SNVs and SVs, to quantify ctDNA burden as a measure of MRD in lymphoma. ddPCR allows specific, sensitive and absolute quantification of targets with a relatively short turn-around time and easy data interpretation (54–57). Conventional ddPCR can distinctly detect one mutational target (singleplex) within each of the two fluorescent channels when using current 2-channel Bio-Rad systems (BioRad, CA, USA). Recently, the amplitude m-ddPCR approach has been shown to enable quantification of ≥2 targets simultaneously within the same channel (43, 44, 58). This is achieved by varying assay concentrations to shift the positive droplet clusters along the amplitude axis, thereby creating space for distinguishing other positive clusters (43, 44, 58). ddPCR can be used to target hotspot mutations, although in most cases the method requires prior knowledge of the genetic aberrations in the tumor. A tumor-informed approach for longitudinal monitoring of treatment response in cancers has been shown to impart higher sensitivity and lower levels of false positive findings (43, 59).

We successfully designed m-ddPCR assays for at least two targets for all 8 patients with informative tumor WGS results. If possible, unique breakpoint sequences of SVs were selected as these are highly specific. We showed that MRD assessment using ddPCR with multiple targets including SVs, such as *IGH::BCL2* translocations, had high detection sensitivity down to 0.0025% with very low false positives. The detection limit of using quantitative PCR and standard NGS-based methods is around 1-5% VAF (60). Even though other studies have shown the potential of targeted, deep-sequencing based methods for quantifying ctDNA burden with high detection sensitivity for MRD in lymphoid neoplasms (16, 19, 47, 60–68), these studies have relied largely on analyzing genomic aberrations in a selected panel of regions and/or clonal IG gene rearrangements in plasma for MRD assessment. However, the costs of ultra-deep sequencing coupled with the need for advanced bioinformatic expertise limits the clinical feasibility of these methods for longitudinal monitoring. Furthermore, deep sequencing methods on cfDNA require extensive optimization and benchmarking of bioinformatic pipelines and tools to call true positive variants among sequencing artefacts and biological noise, such as non-tumor-related, somatic variants associated with clonal hematopoiesis of indeterminate potential (CHIP) (69).

Total cfDNA copies/ml and ctDNA levels measured by m-ddPCR in diagnostic plasma suggested correlation with clinical parameters such as serum LDH, staging, tumor bulk, and the COO classification in our cohort. Although we refrained from statistical testing owing to the small number of patients per subgroup, the trends were in agreement with previous findings reported in lymphoma, with higher total cfDNA and ctDNA levels at diagnosis correlating with clinical parameters associated with poor patient outcome (18, 47, 61–63, 66, 70–72). Furthermore, higher ctDNA levels at diagnosis in our cohort were observed in patients with PR at interim evaluation, previously reported as a strong prognostic indicator (73, 74). However, further investigation and validation in larger cohorts is required to assess the prognostic and predictive significance of the diagnostic ctDNA burden in lymphoma.

Plasma cfDNA analysis correlated to a large extent with radiological evaluations during primary and relapse treatment, as has been previously reported (17, 65, 75). Three discordant results were at interim evaluation of Pat2, Pat3 and Pat5 where no ctDNA was detected; however, CT reported PR. At the end of treatment, all three patients remained ctDNA negative and PET-CT analysis showed CR. It is known that CT cannot always distinguish between viable and non-viable residual tumor, therefore PET-CT is preferred for a more accurate treatment evaluation.

Rapid clearance of MRD during first-line treatment has been correlated with prognosis in lymphoma (76). In our cohort, Pat5 was one of the patients with MRD detected by plasma analysis after the first cycle of primary treatment and who later relapsed. This patient had negative ctDNA at end of primary treatment, but the follow-up samples showed positive ctDNA 25 weeks earlier than the clinical manifestation of the relapse. Our study thus asserts the significance of serial sampling and quantification of MRD after end of primary treatment for early detection of recurrent disease. Previous studies have also shown that ctDNA detection after frontline treatment was significantly indicative of disease progression and identified recurrence preceding clinical manifestation of relapse (47, 62, 65, 75). In the other relapsed patient, Pat1, plasma samples were neither collected after first cycle of primary treatment nor between the first follow-up (~13 weeks after final evaluation of primary treatment) and the clinical detection of relapse (~2 years later). This was due to covid-19 pandemic-related restrictions on clinical visits for asymptomatic patients. Therefore, the impact of ctDNA analysis for early detection of relapse could not be evaluated in Pat1.

To conclude, we have, to the best of our knowledge, presented the first-of-its kind, clinically feasible WGS-informed, multi-targeted ddPCR-based approach, targeting both SNVs and SVs. We have shown the clinical potential of this sensitive, personalized approach for monitoring MRD in lymphoma patients using serially collected plasma samples. As the sequencing cost per genome is continuously decreasing, we envision that in the coming years, WGS can be implemented in the clinical diagnostics of lymphoma, providing a comprehensive characterization of all genomic aberrations. This will not only enable improved genetic classification of lymphoma subtypes at diagnosis, but also resolve genomic breakpoints of driver SVs that can then be utilized as robust, sensitive targets for personalized longitudinal monitoring using liquid biopsies.

## Data availability statement

The datasets presented in this study can be found in online repositories and are available upon request from the authors. The names of the repository/repositories and accession number(s) can be found below: https://doi.org/10.17044/scilifelab.22193854.v1.

## Ethics statement

The studies involving human participants were reviewed and approved by Stockholm Regional Ethical committee (2017/2538-31). The patients/participants provided their written informed consent to participate in this study.

## Author contributions

The study was designed and conceptualized by ZH, ET, RR, KES and TW. Laboratory tasks were performed by ZH, CJ, AL and AG. Bioinformatic analyses were conducted by ZH, BT, MH, AJ, AS, and LR. Patient clinical data was collected and compiled by TW, LD, AK, HA, KS, BS, GR and KES. The manuscript was drafted by ZH, ET, TW, KES, RR, while all other coauthors equally contributed to proofreading the final version. All authors contributed to the article and approved the submitted version.
