# Supplementary material for: Whole-genome informed circulating tumor DNA analysis by multiplex digital PCR for disease monitoring in B-cell lymphomas: a proof-of-concept study
Source: Front Oncol. 2023 Jun 2;13:1176698. doi: 10.3389/fonc.2023.1176698 (PMC10272573; doi:10.3389/fonc.2023.1176698)
Supplement: Supplementary file 1 [file DataSheet_1.pdf]

Supplementary Figures

Whole-genome informed circulating tumor DNA analysis by multiplex digital PCR for disease monitoring in B-cell lymphomas: a proof-of-concept study

Zahra Haider[1]*, Tove Wästerlid[2,3], Linn Deleskog Spångberg[2], Leily Rabbani[1], Cecilia Jylhä[1,4], Birna Thorvaldsdottir[1], Aron Skaftason[1], Hero Nikdin Awier[4], Aleksandra Krstic[1,4], Anna Gellerbring[5], Anna Lyander[5], Moa Hägglund[5], Ashwini Jeggari[5], Georgios Rassidakis[6,7], Kristina Sonnevi[2,3], Birgitta Sander[8], Richard Rosenquist[1,4,9], Emma Tham[1,4]†, Karin E. Smedby[2,3]†

* Corresponding Author: Zahra Haider, zahra.haider@ki.se
†These authors share senior authorship.

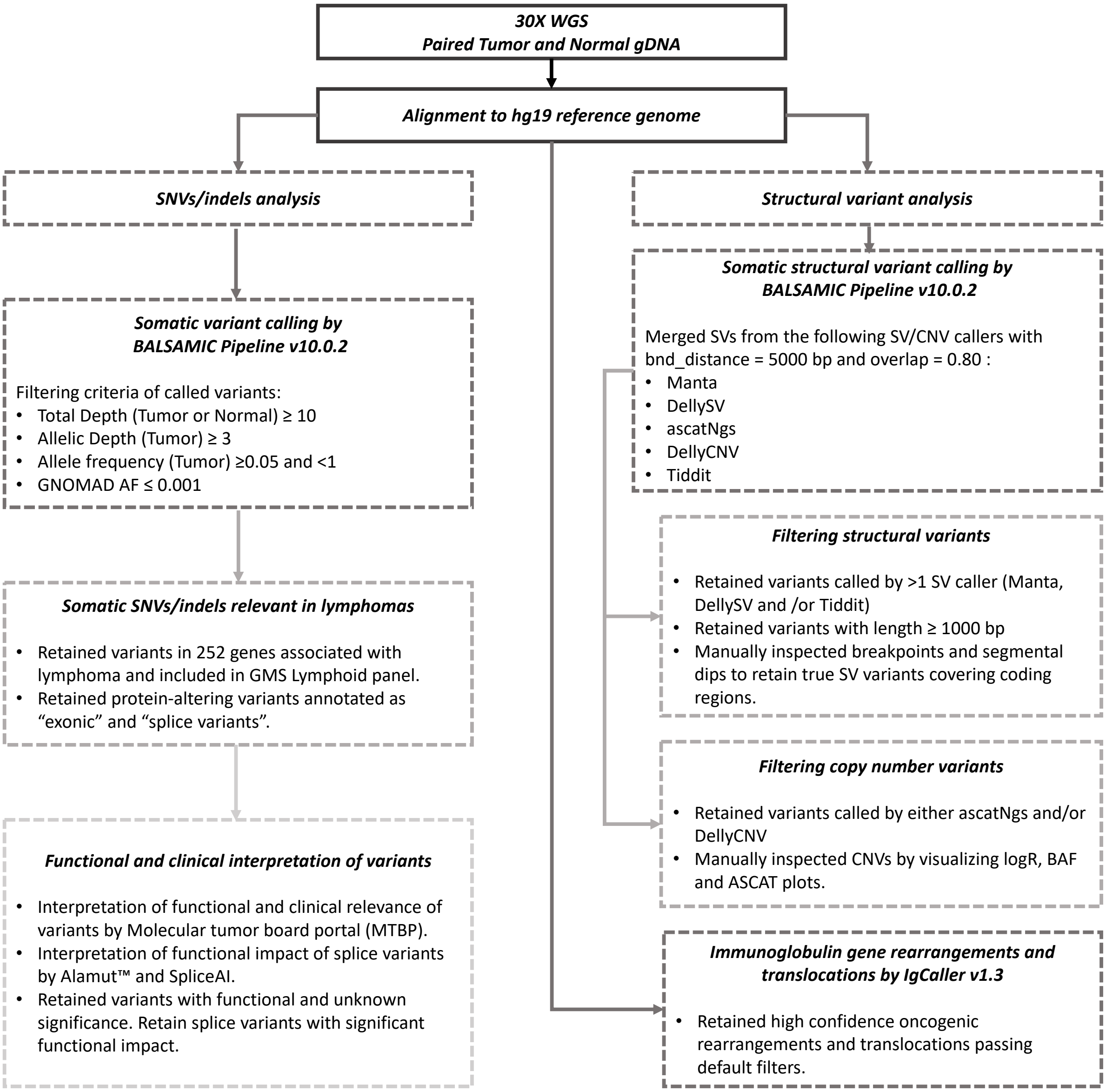

Figure S1. Comprehensive genomic profiling in lymphomas by whole-genome sequencing. Whole genome sequencing analysis workflow for single nucleotide variants (SNVs), small insertions and deletions (indels), large structural variants (SV) and copy number variants (CNV) calling, including filtering and variant classification strategies to retain somatic aberrations relevant to lymphoma pathogenesis. Reference numbers are stated in paranthesis. BAF, B-allele frequency.

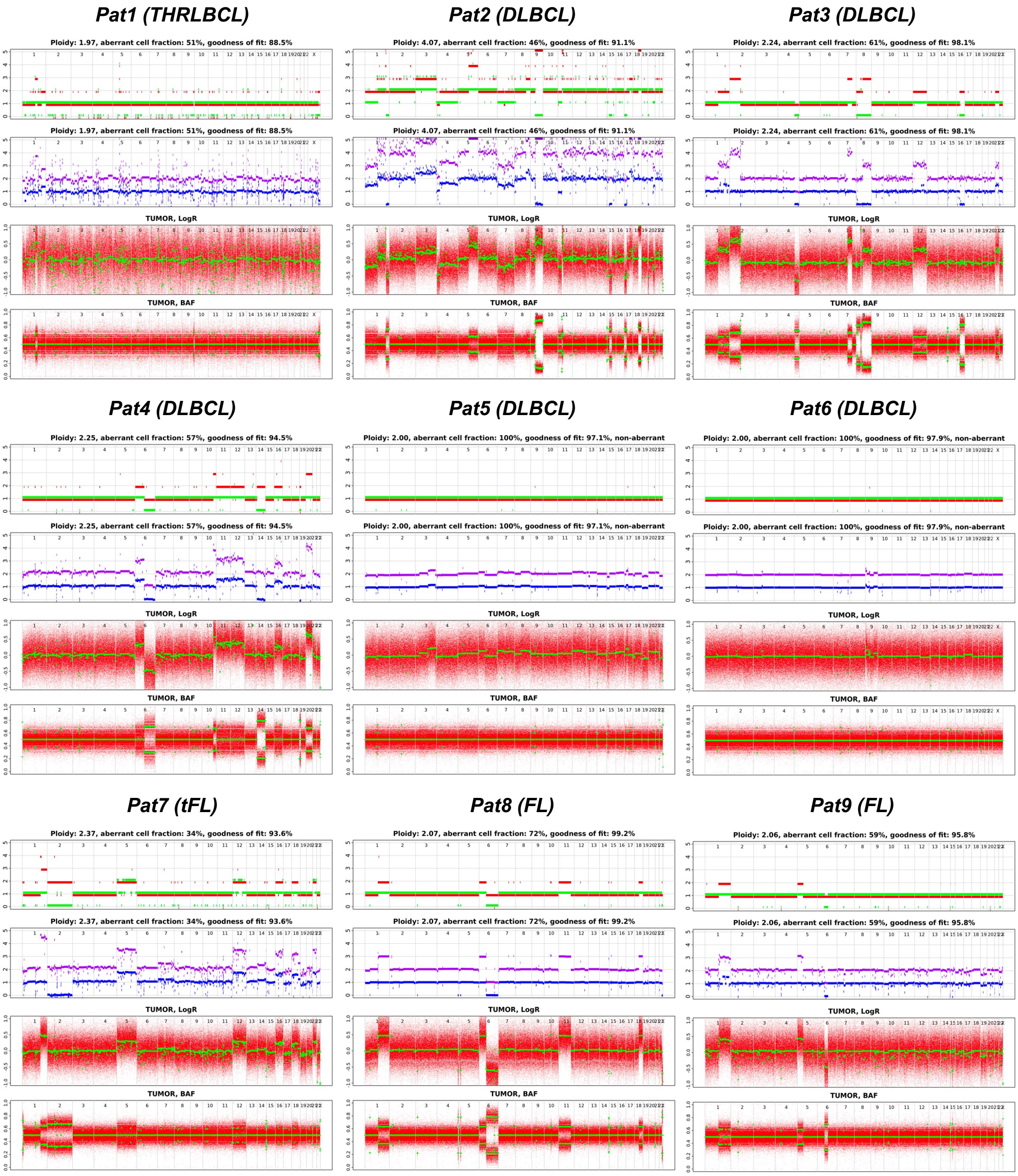

Figure S2. Somatic whole genome copy number variant profiles in lymphoma. Copy number profiles by ascatNgs analysis of tumor samples from 9 patients, showing (bottom to top) i) segmented B-allele fraction (BAF), ii) segmented LogR, iii) ASCAT raw profile with total (purple) and minor allele copy number (blue) and iv) ASCAT profile with minor allele copy number (green) and estimated total copy number (red) along genomic loci (χ-axis), with ploidy estimates.

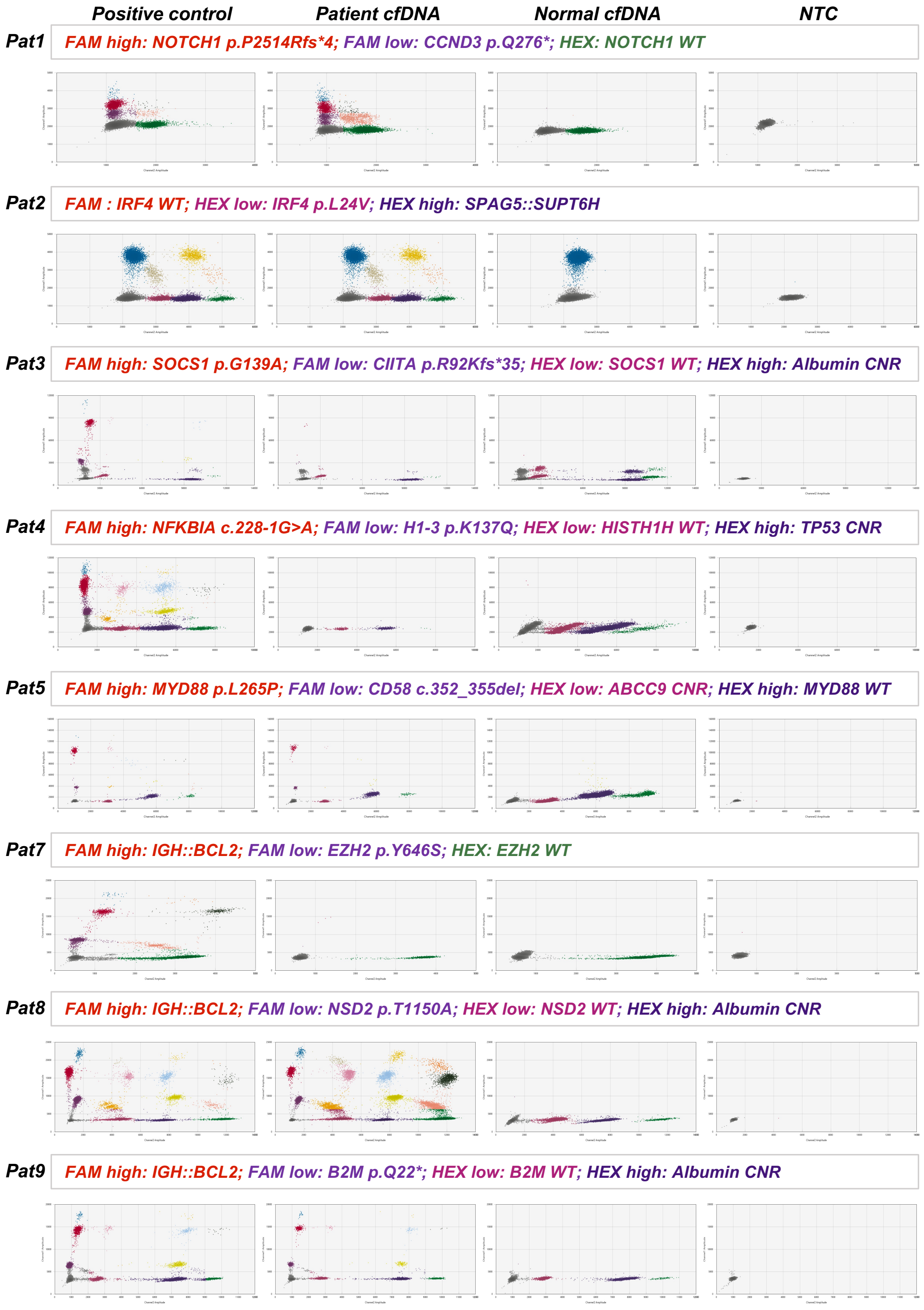

Figure S3: Optimized patient-specific multiplex ddPCR assays used for plasma analysis. Representative 2D amplitude plots obtained from QuantaSoft™ Analysis Pro (Bio-Rad) showing optimized patient-specific multiplex ddPCR assays. Assays were tested in multiple wells containing genomic DNA from diagnostic tumor tissue (positive control, 3 wells), cfDNA from diagnostic plasma sample (patient cfDNA, 3 wells), and normal cfDNA from healthy donors (9-12 wells) and non-template control (NTC, 3 wells).

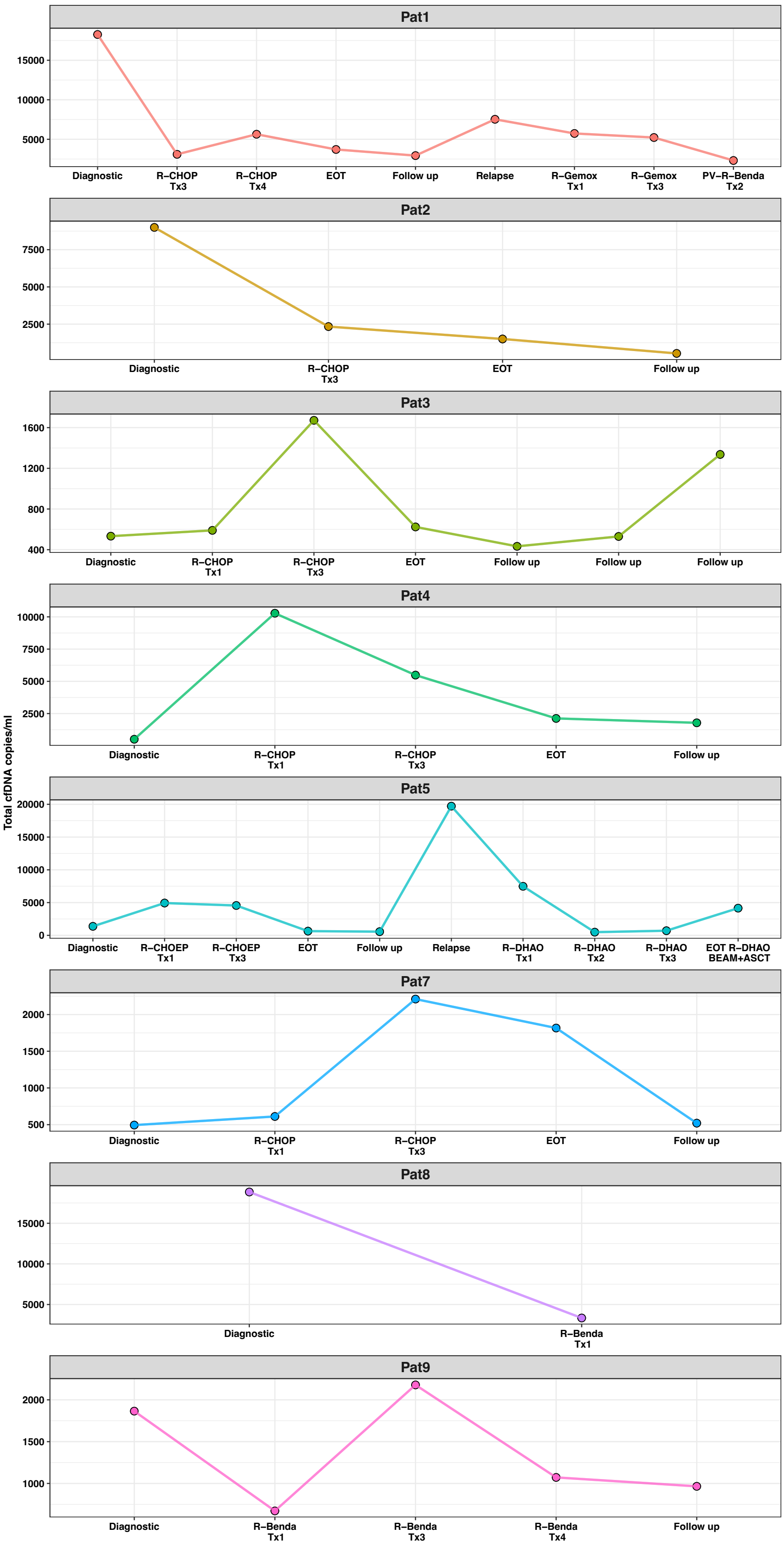

Figure S4. Total cfDNA copies/ml in plasma at diagnosis and follow-up quantified by multiplex ddPCR. Tx, treatment cycle; EOT, end of treatment.

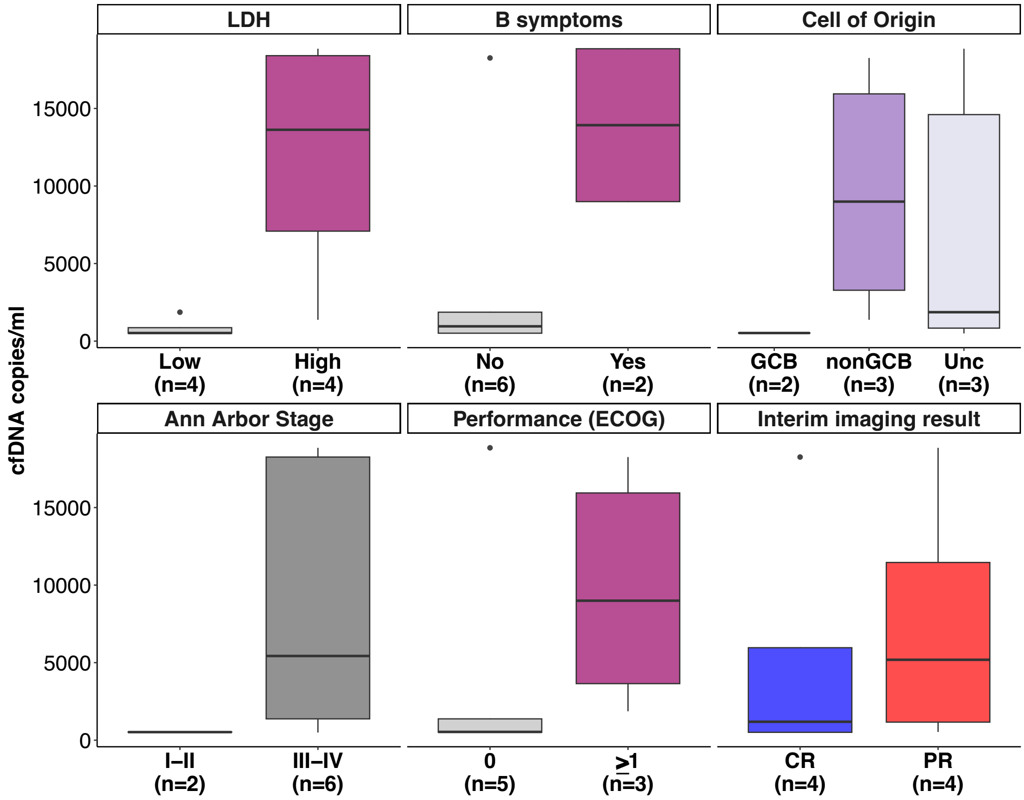

Figure S5. Correlation of total cfDNA copies/ml analyzed in diagnostic plasma samples with baseline clinical characteristics and interim imaging results.
